# Innovative aspects of tooth ultrastructure and ultra-chemistry: unraveling of caries mechanisms development prevention strategies

**DOI:** 10.1186/1878-5085-5-S1-A124

**Published:** 2014-02-11

**Authors:** Anatoly A Kunin, Irina A Belenova

**Affiliations:** 1 Professors, Dr. Med. Sc., Voronezh N.N. Burdenko State Medical Academy, Therapeutic Dentistry Department, Voronezh, Russia

## 

Investigation of the structure and morph-chemistry of enamel and dentin should ultimately lead to development of effective caries prevention strategies. Accordingly, we have studied the nanostructure and morph-chemistry of teeth utilizing X-ray microanalysis, scanning electron microscopy and atomic force microscopy and tunneling. The results suggest that the surface of primary and permanent teeth enamel is not as smooth as previously considered. Rather enamel surface has apertures which are conducive to realization of morphological and chemical processes. Detailed examination of the enamel surface reveals that most of anatomical defects/holes do not end blindly; rather, they penetrate the enamel surface (resembling "tunnels"), branch and intersect each other to form enamel "bridges" in dentin. Examination of enamel using atomic-powered and tunnel microscopy not only confirm the presence of these nanostructures, - but also help discern more refined characteristics. Consequently, it appears that the tunnels intersect one another at 90 degrees angle. The morph-chemical examination of enamel reveals differences in the structure of intact compared to carious enamel and dentin. Calcium and phosphorus - are the main structural elements of enamel and dentin. The amount of potassium, sulfur and chlorine increases respectively in caries pathology, while the concentration of sodium, silicon, and especially magnesium decreases. The same pattern is observed for dentin. These observations suggest that high concentration of magnesium increases, while chlorine and sulfur decrease the effectiveness of the protective properties of enamel thereby leading to alteration of caries resistance. Subsequently, we examined teeth obtained from animals. In beaver’s enamel, the content of magnesium is higher (seven fold) compared to those of human. The concentration of chlorine and sulfur are higher in people’s enamel that confirms our previous researches concerning the impact of these elements on the caries resistance level of enamel. Differences in microelements structure, the prevalence of magnesium in beaver’s enamel, determined the differences in microhardness of the enamel. The content of the elements is 1.5 times higher in tooth enamel of beavers than humans*.* In this case, calcium-phosphorus index of animals’ enamel, with high caries resistance, have no significant differences with the calcium-phosphorus index of human enamel rather, it is even lower.

In conclusion, the novel discovery of structural formations and the morph-chemical structure of human and animal enamel may ultimately lead to identification of a new approach to caries prevention and development of new, more effective formulation of oral hygiene products.

